# Abdominal girth has a strong correlation with actual and ultrasound estimated epidural depth

**DOI:** 10.3906/sag-1902-115

**Published:** 2019-12-16

**Authors:** Mehmet CANTÜRK, Nazan KOCAOĞLU, Meltem HAKKI

**Affiliations:** 1 Department of Anesthesiology and Reanimation, Ahi Evran University Training and Research Hospital, Kırşehir Turkey; 2 Department of Anesthesiology and Reanimation, Faculty of Medicine, Balıkesir University, Balıkesir Turkey

**Keywords:** Abdominal girth, anesthesia, epidural, ultrasound

## Abstract

**Background/aim:**

This study aimed to assess the correlations of actual epidural depth (ND) and ultrasound estimated epidural depth in the paramedian sagittal oblique plane (ED/PSO) and transverse median plane (ED/TM) with the abdominal girth (AG), body mass index (BMI), and weight of patients.

**Materials and methods:**

One hundred and thirty patients of either sex scheduled for unilateral inguinal hernia repair were enrolled. ED/PSO and ED/TM were assessed with a 2–5 MHz curved array probe at the L3–4 intervertebral space. The epidural needle was marked with a sterile marker upon locating the epidural space. The ND was assessed by measuring the distance from the sterile marker to the tip of the epidural needle with a linear scale. Anthropometric measures of the patients were recorded.

**Results:**

ED/PSO was 49.6 ± 7.9 mm, ED/TM was 49.5 ± 7.9 mm, and ND was 50.0 ± 8.0 mm. AG was 99.8 ± 12.9 cm. The Pearson correlation coefficient between ND and ED/PSO was 0.997 and with ED/TM was 0.996 (P < 0.001 for both). Pearson correlation coefficients for ND with AG, BMI, and weight were 0.757, 0.547, and 0.638, respectively (P < 0.001 for all).

**Conclusion:**

AG, weight, and BMI have strong correlations with ND.

## 1. Introduction

Combined spinal epidural anesthesia (CSE) is widely used for inguinal hernia repair surgeries. The precise location of the epidural needle navigated by ultrasound has a crucial role in the success rate of CSE, providing the needle trajectory and the exact level of desired intervertebral space [1]. Preprocedural ultrasound scanning of the vertebral column may facilitate the insertion of the epidural needle during CSE [2]. 

Former studies assessing the depth of lumbar epidural space with ultrasound used the transverse median plane (TM) and reported positive correlations with the actual epidural depth (ND) [3–5]. In a limited number of studies conducted with parturient patients, longitudinal plane ultrasound examination was combined with TM to estimate the ND [1,2,6]. Grau et al. [7] reported paramedian access to the epidural space as the optimal window for ultrasound imaging. The accuracy of paramedian epidural depth assessment was also reported for thoracic epidural insertions [8,9]. In 2014, Kim et al. [10] reported the superiority of the paramedian sagittal approach during pararadicular injection at the lumbar spine.

Several patient characteristics and anthropometric measurements were assessed to predict ND [3,11–16]. Of these parameters, weight and body mass index (BMI) were reported to have a strong correlation with ND. 

In the current study, we assessed the correlation of ND with abdominal girth (AG), weight, height, and body mass index (BMI) to derive a mathematical predictor formula for ND at the L3–4 level.

## 2. Materials and methods

### 2.1. Ethics and patients

After approval from the institutional review board and local ethics committee (2017-20/241, 26/12/2017), the study was prospectively registered with the Australian New Zealand Clinical Trials Registry (ACTRN12618000586213) and signed informed consent was obtained from 124 patients of ASA physical status I–III, aged 18–80 years and scheduled for elective unilateral inguinal hernia repair surgery. Patients were excluded from the study if they met one or more of the following criteria: prior spine or spinal canal surgery, vertebral canal deformities, coagulopathy or usage of anticoagulant drug medication, pregnancy, ASA physical status of >III, any neurological disease, refusal to participate in the study, infection at the site of epidural injection, and other contraindications for neuraxial blocks.

Patients were monitored by electrocardiogram, pulse oximeter, and noninvasive blood pressure. An intravenous line was secured on the dorsum of the left hand for intravenous hydration and medication.

Data on age, weight, height, and BMI were recorded in the medical fields of the patients during their preoperative visit. Abdominal girth was measured by the same investigator at the level of the umbilicus in the transverse plane with a measuring tape while the patients in a sitting position and the data were recorded.

### 2.2. Ultrasound scanning

Patients were placed in a sitting position on the operating table with a stool under their feet. Patients’ knees, hips, and neck were flexed. Ultrasound imaging was done before sterile draping by the same investigator using a portable ultrasound (Esaote MyLab30, Florence, Italy). The 2–5 MHz curved array probe was first placed in the midline sagittal plane to determine the hyperechoic shadow of the sacrum. The probe was then advanced cranially to determine the L3–4 intervertebral space. With the location of the intervertebral space in the center of the ultrasound probe, the skin was marked on both sides of the probe in the midline. The skin marks were elongated as a horizontal line, and the probe was located on this line and inclined in a cephalad or caudal manner to obtain the acoustic window of the L3–4 intervertebral space. The midpoint of the probe was marked on the skin in the sagittal plane, and these two skin marks were elongated in the sagittal plane. The intersection of the horizontal and vertical lines determined the epidural needle insertion point.

Paramedian sagittal oblique (PSO) plane measurements of epidural depth were performed initially. The curved array probe of the ultrasound was placed 1–2 cm lateral to the midline on the horizontal plane at the level of the L3–4 intervertebral space. The probe was tilted through the midline to obtain the PSO view of the vertebral canal. The image on the ultrasound screen was frozen and recorded for measurement of ultrasound-estimated epidural depth in the paramedian sagittal oblique plane (ED/PSO). The distance from the skin to the posterior border of the ligamentum flavum–posterior dura complex (posterior complex) was accepted as the ED/PSO.

With the completion of PSO scanning of the vertebral canal, the curved array ultrasound probe was oriented in the transverse plane at the L3–4 intervertebral space in the midline and the screen was frozen for the measurement of ultrasound-estimated epidural depth in the transverse median plane (ED/TM). 

### 2.3. Combined spinal-epidural anesthesia and actual epidural depth

The skin was cleansed of ultrasound gel and prepared with an antiseptic solution. The patient position was the same during the ultrasound scanning and the CSE procedure. CSE was performed from the previously marked insertion point determined by ultrasound. Following sterile draping and local anesthetic infiltration of skin and subcutaneous tissue with 5 mL of 1% lidocaine, the epidural needle of the combined spinal-epidural set (B. Braun Melsungen AG, Melsungen, Germany) was advanced until it reached the interspinous ligament. The guide of the epidural needle was withdrawn, and the air-filled sterile syringe was locked to the hub of the epidural needle. The epidural needle was advanced until the identification of epidural space with the loss of resistance to air technique with the midline approach. The spinal needle was introduced with the needle-through-needle technique. After observing the free flow of clear cerebrospinal fluid, 3 mL of 0.5% hyperbaric bupivacaine was injected into the intrathecal space. The spinal needle was removed, and the epidural catheter was advanced by 5 cm to the epidural space. The epidural needle was marked with a sterile marker on the skin before it was withdrawn. Actual epidural depth was measured on the epidural needle between the sterile marker and the tip of the needle with a linear scale that had millimeter calibration on it. The epidural catheter was fixed, and the patient was turned to a supine position. The spread of spinal anesthesia was assessed with loss of pain sensation by the pin-prick test. Surgery was commenced when the level of spinal block reached the T6 dermatome level.

The primary outcomes of the study were the determination of ND, ED/PSO, and ED/TM. Secondary outcomes of the study were the measurement of AG, weight, height, and BMI of the patients. The objective of the study was to assess the correlation of the ND with AG, weight, height, and BMI of the patients and determine the correlation of ND with ED/PSO and ED/TM in patients scheduled for elective unilateral inguinal hernia repair surgery with CSE. A mathematical equation to estimate ND was generated from the regression analysis model performed with IBM SPSS 21.0 (IBM Corp., Armonk, NY, USA). Keeping ND as the dependent variable and age, weight, height, BMI, and AG as independent variables in the linear stepwise regression analysis model, a mathematical formula was derived to estimate ND. 

### 2.4. Statistical analysis

IBM SPSS 21.0 was used for the analysis of data in the current study. Descriptive data were expressed as means and standard deviations for continuous parameters and as percentages for nominal parameters. A bivariate linear correlation analysis (Pearson correlation analysis) was used to test the correlations between ND, ED/PSO, and ED/TM and the age, weight, height, AG, and BMI of patients. The correlations of ND with ED/PSO and ED/TM were also analyzed with Pearson correlation analysis. Linear regression analysis was used with a stepwise method to detect the correlation of ND, ED/PSO, and ED/TM with patients’ age, weight, height, AG, and BMI. Skin-to-epidural depth was determined as the dependent variable in all three linear regression models (ND, ED/PSO, and ED/TM) and the patients’ characteristics and anthropometric measures were the independent variables. P < 0.05 was considered statistically significant.

The sample size of the study was calculated using the data obtained from the preliminary results of the study (AG = 99.9 ± 10.7 cm, ED/PSO = 49.9 ± 6.7 mm, ED/TM = 49.8 ± 6.8 mm, ND = 50.2 ± 6.8 mm) with G*Power 3.1.9.2. The minimum required sample size (n) was 120 to detect the desired statistical power level of 0.95 and P-value of 0.05.

## 3. Results

**Figure 1:**
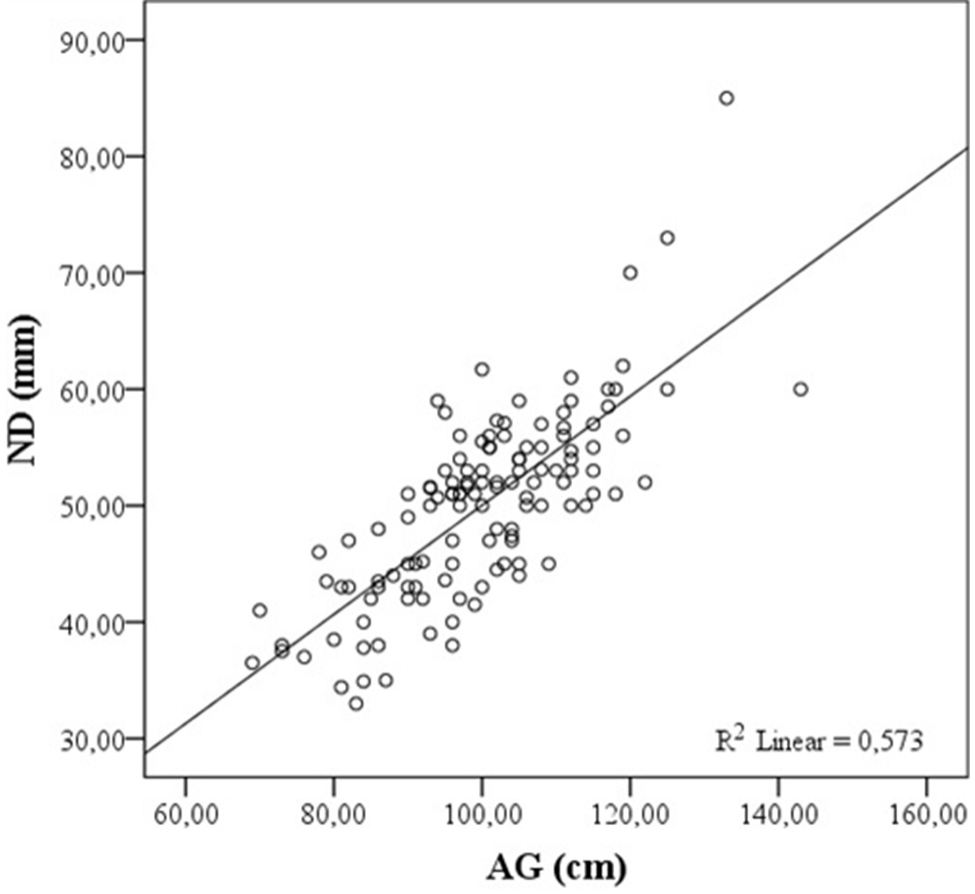
Correlation of AG with ND. ND: Actual epidural depth, AG: abdominal girth.

**Figure 2:**
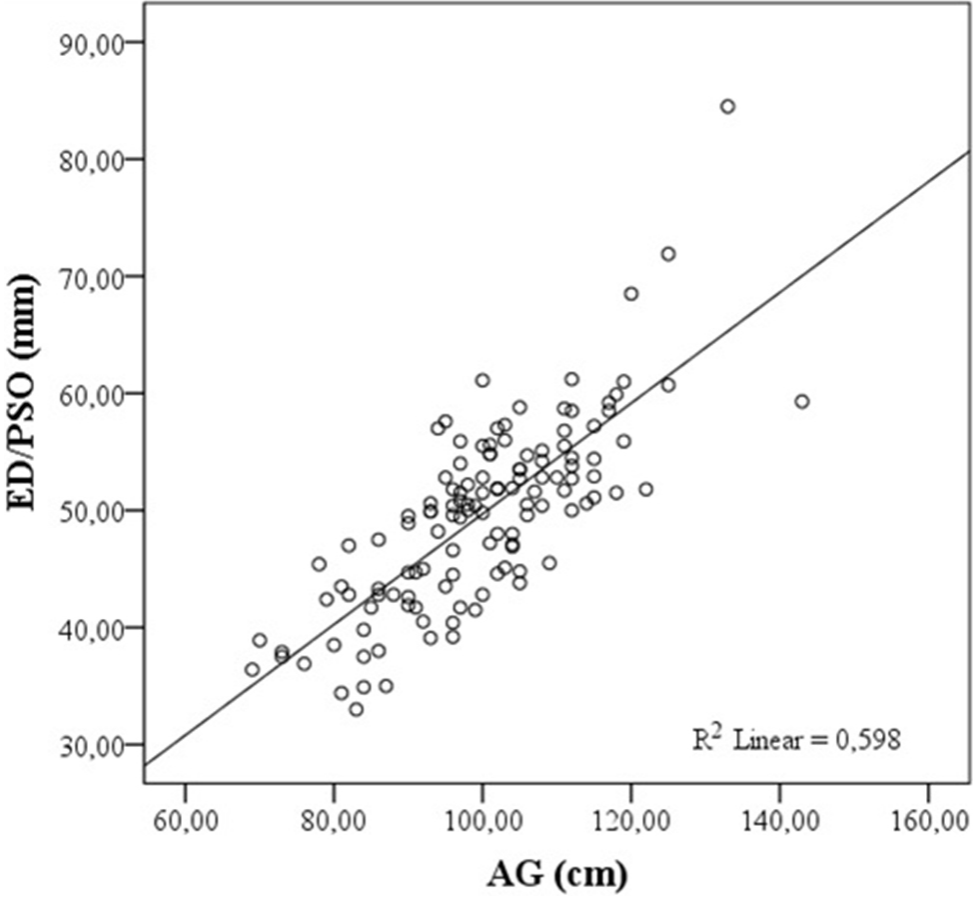
Correlation of AG with ED/PSO. ED/PSO: Ultrasound estimated skin to epidural depth in paramedian sagittal oblique plane, AG: abdominal girth.

**Figure 3:**
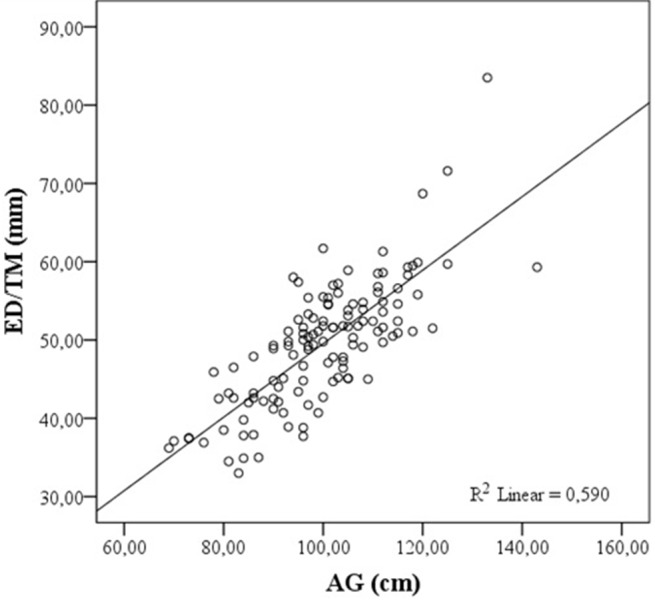
Correlation of AG with ED/TM. ED/TM: Ultrasound estimated skin to epidural depth in transverse median plane, AG: abdominal girth.

A total of 130 patients were enrolled in the study, and there were six dropouts (two patients refused to be involved in the study, anticoagulant medication was prescribed for one patient, and three patients wanted to postpone their surgical procedures to future dates). Data obtained from 124 patients were thus included in the statistical analysis of the current study. Patient characteristics are presented in Table 1. Mean patient age was 50.12 ± 14.72 years, mean patient height was 169.45 ± 9.05 cm, mean patient weight was 78.49 ± 12.40 kg, and the mean BMI was 27.51 ± 4.80 kg/m2. Thirty-seven patients were female (29.8%) and 87 were male (70.2%). Sixty-one patients were of ASA physical status I (49.2%), 49 of ASA physical status II ((39.5%), and 14 of ASA physical status III (11.3%). Mean ND was 49.96 ± 8.01 mm, mean ED/PSO was 49.63 ± 7.91 mm, mean ED/TM was 49.46 ± 7.90 mm, and mean AG was 99.8 ± 12.9 cm. ND was strongly correlated with the AG (r = 0.757, 95% CI: 0.685–0.814, P < 0.001), weight (r = 0.638, 95% CI: 0.526–0.725, P < 0.001), and BMI (r = 0.547, 95% CI: 0.394–0.687, P < 0.001) of the patients. Correlation coefficients of ND, ED/PSO, and ED/TM with age, height, weight, BMI, and AG are presented in Table 2. The correlations of AG with ND, ED/PSO, and ED/TM are presented in Figures 1–3. Keeping ND as a dependent variable and age, weight, height, BMI, and AG as independent variables in the linear stepwise regression analysis model, a mathematical formula was derived estimating ND as a = 0.469b + 3.17, where a = ND in millimeters and b = AG in centimeters. Keeping ED/PSO as the dependent variable with the same independent variables, the mathematical equation to estimate ED/PSO was a = 0.473b + 2.43, where a = ED/PSO in millimeters and b = AG in centimeters. Keeping ED/TM as the dependent variable with the same independent variables, the mathematical equation to estimate ED/TM was a = 0.470b + 2.58, where a = ED/TM in millimeters and b = AG in centimeters.

**Table 1:** Patient characteristics.

ASA I / II / III, n (%)	61 (49.2) / 49 (39.5) / 14 (11.3)
Sex, F/M, n (%)	37 (29.8) / 87 (70.2)
Age, years	50.12 ± 14.72
Weight, kg	78.50 ± 12.40
Height, cm	169.45 ± 9.06
BMI, kg/m2	27.52 ± 4.80
AG, cm	99.85 ± 12.94
ED/PSO, mm	49.63 ± 7.91
ED/TM, mm	49.46 ± 7.91
ND, mm	49.96 ± 8.01

ASA: American Society of Anesthesiologist physical status, F: female, M: male, BMI: body mass index, AG: abdominal girth, ED/PSO: ultrasound estimated skin to epidural depth in paramedian sagittal oblique plane, ED/TM: ultrasound estimated skin to epidural depth in the transverse median plane. ND: actual skin to epidural depth. Values are expressed as mean ± standard deviation except for ASA and sex.

**Table 2:** Correlation of patient characteristics with ND, ED/PSO, and ED/TM

	ND			ED/PSO			ED/TM		
	r	95% CI	P	r	95% CI	P	r	95% CI	P
Age (years)	–0.123	–0.023 to 157	0.797	–0.012	–0.197 to 0.171	0.896	–0.013	–0.195 to 166	0.883
Weight (kg)	0.638	0.526 to 0.725	<0.001	0.648	0.541 to 0.731	<0.001	0.654	0.545 to 0.737	<0.001
Height (cm)	0.025	–0.164 to 0.215	0.784	0.021	–0.169 to 0.209	0.817	0.021	–0.164 to 0.208	0.816
BMI (kg/m^2^)	0.547	0.394 to 0.687	<0.001	0.558	0.406 to 696	<0.001	0.564	0.415 to 700	<0.001
AG (cm)	0.757	0.685 to 0.814	<0.001	0.773	0.708 to 0.827	<0.001	0.768	0.699 to 0.825	<0.001

ND: Actual epidural depth, ED/PSO: ultrasound estimated skin to epidural depth in paramedian sagittal oblique plane, ED/TM: ultrasound estimated skin to epidural depth in transverse median plane, BMI: body mass index [weight / (height)^2^], AG: abdominal girth, r: Pearson correlation coefficient, P: statistical significance, 95% CI: 95% confidence interval for Pearson correlation coefficient (r).

## 4. Discussion

Our study demonstrated a strong correlation between ND and AG, weight, and BMI in patients scheduled for elective unilateral inguinal hernia repair surgery with CSE. We have also demonstrated a strong correlation between ND, ED/PSO, and ED/TM.

Former studies reported a strong correlation between ND and ED/TM [2–6,8,17]. The results of the current study are in line with the previous studies. The correlation of ND with ED/PSO, however, has not been assessed widely in the lumbar region. Sahota et al. [18] assessed the correlation of ED/TM with ED/PSO in 60 parturient patients. In that study, they concluded that both planes could be used interchangeably to estimate epidural depth during midline neuraxial punctures. The usefulness of PSO ultrasound scanning was reported in two studies during thoracic epidural insertion [8,9]. Both studies presented a strong correlation between the ND and the ED/PSO at the thoracic level. Our study presented a strong correlation between ND and ED/PSO (r = 0.997, P < 0.001) at the lumbar region. We conducted our study in a nonpregnant patient population, and the ultrasound estimated epidural depth measurements were at the level of the L3–4 intervertebral space. Although the patient population in the current study was different from that of the study by Sahota et al. [18] and the vertebral level was different from those of the studies by Khemka et al. [8] and Salman et al. [9], our study results were in accordance with those studies to suggest that ED/PSO reliably estimates the ND.

Correlation of weight and BMI with ND was previously reported, and the correlation coefficients ranged from 0.597 to 0.762 for BMI and ND in these studies [11,12,14,15]. The correlation coefficient between ND and BMI in the current study was also in accordance with those former studies. 

One of the novel findings presented in our study was the correlation of ND with the AG of the patients. In the linear regression model, we derived a mathematical formula estimating ND as a = 0.469b + 3.17 where a = ND in millimeters and b = AG in centimeters. Assuming a patient with AG measurement of 99.84 cm (this is the mean AG value in the current study), estimated ND would be 0.469 × 99.84 + 3.17 = 49.97 mm. The mean ND in the current study was 49.96 mm. The AG-based equation derived from the linear regression model reliably estimates the ND. When ultrasound guidance is not readily present in the operating room, clinicians can make a reliable estimation of the epidural depth with the equation presented above. However, we have to remind clinicians not to discard the loss of resistance technique while performing epidurals with the guidance of ND estimates obtained from the equation suggested in the current study.

The main limitation of the current study was patients of ASA physical status >III, patients younger than 18 years and older than 80 years, and parturient patients not being included in the study population. 

In conclusion, both PSO and TM ultrasound scanning provides a reliable estimate for ND. When ultrasound is not available for preprocedural assessment of epidural depth because of being sophisticated and expensive equipment, the AG-based mathematical equation suggested in the current study may provide a reliable estimate of the ND for the clinicians performing epidurals.
